# Editorial: Advancements in Technology-Based Assessment: Emerging Item Formats, Test Designs, and Data Sources

**DOI:** 10.3389/fpsyg.2019.03047

**Published:** 2020-01-20

**Authors:** Frank Goldhammer, Ronny Scherer, Samuel Greiff

**Affiliations:** ^1^Educational Quality and Evaluation, DIPF - Leibniz Institute for Research and Information in Education, Frankfurt, Germany; ^2^Centre for International Student Assessment (ZIB), Frankfurt, Germany; ^3^Centre for Educational Measurement (CEMO), University of Oslo, Oslo, Norway; ^4^Cognitive Science & Assessment, University of Luxembourg, Esch-sur-Alzette, Luxembourg

**Keywords:** technology-based assessment, item design, test design, automatic scoring, process data, assessment of/for learning

Technology has become an indispensable tool for educational and psychological assessment in today's world. Individual researchers and large-scale assessment programs alike are increasingly using digital technology (e.g., laptops, tablets, and smartphones) to collect behavioral data beyond the mere correctness of item responses. Along these lines, technology innovates and enhances assessments in terms of item and test design, methods of test delivery, data collection and analysis, and the reporting of test results.

The aim of this Research Topic is to present recent developments in technology-based assessment and in the advancements of knowledge associated with it. Our focus is on cognitive assessments, including the measurement of abilities, competences, knowledge, and skills, but also includes non-cognitive aspects of assessment (Rausch et al.; Simmering et al.). In the area of (cognitive) assessments, the innovations driven by technology are manifold, and the topics covered in this collection are, accordingly, wide and comprehensive: Digital assessments facilitate the creation of new types of stimuli and response formats that were out of reach for assessments using paper; for instance, interactive simulations may include multimedia elements, as well as virtual or augmented realities (Cipresso et al.; de-Juan-Ripoll et al.). These types of assessments also allow for the widening of the construct coverage in an assessment; for instance, through stimulating and making visible certain problem-solving strategies that represent new forms of problem solving (Han et al.; Kroeze et al.). Moreover, technology allows for the automated generation of items based on specific item models (Shin et al.). Such items can be assembled into tests in a more flexible way than what is possible in paper-and-pencil tests and can even be created on the fly; for instance, tailoring item difficulty to individual ability (adaptive testing) while assuring that multiple content constraints are met (Born et al.; Zhang et al.). As a requirement for adaptive testing, or to lower the burden of raters who code item responses manually, computers enable the automatic scoring of constructed responses; for instance, text responses can be coded automatically by using natural language processing and text mining (He et al.; Horbach and Zesch).

Technology-based assessments provide not only *response data* (e.g., correct vs. incorrect responses) but also *process data* (e.g., frequencies and sequences of test-taking strategies, including navigation behavior) that reflect the course of solving a test item and gives information on the path toward the solution (Han et al.). Process data, among others, have been used successfully to evaluate and explain data quality (Lindner et al.), to define process-oriented latent variables (De Boeck and Scalise), to improve measurement precision, and to address substantial research questions (Naumann). Large-scale result and process data also call for data-driven computational approaches in addition to traditional psychometrics and new concepts for storing and managing data (von Davier et al.).

The contributions of this Research Topic address how technology can further improve and enhance educational and psychological assessment from various perspectives. Regarding educational testing, not only is research presented on the assessment *of* learning, that is, the summative assessment of learning outcomes (Molnár and Csapó), but a number of studies on this topic also focus conceptually and empirically on the assessment *for* learning, that is, the formative assessment providing feedback to support the learning process (Arieli-Attali et al.; Blaauw et al.; Csapó and Molnár; den Ouden et al.; Kroeze et al.).

Table 1 gives an overview of all the papers included in this Research Topic and summarizes them with respect to their key features. Reflecting the scope of the Research Topic, we used four major categories to classify the papers: (1) papers focusing on the use of new data types and sources, (2) innovative item designs, (3) innovative test designs, and (4) statistical approaches. We refrained from multiple category assignments of papers, which was possible, and focused on their core contribution. The papers' key findings and advancements impressively represent the current state-of-the-art in the field of technology-based assessment in (standardized) educational testing, and, as topic editors, we were happy to receive such a great collection of papers with various foci.

**Table 1:** Overview of the papers.

**References**	**Area(s) of advancement**	**Data types**	**Statistical approach**	**Assessment purpose (of/for learning)**	**Assessment domains**	**Key finding and advancement**
**Focus on new data types and sources**						
Blaauw et al.	Computerized assessment of learning with multiple informants	Survey responses, platform user data	Descriptive approach	For	Vocational education	Multi-informant time-series data can inform the success of educational interventions to support students at risk
De Boeck and Scalise	Log-file and performance data to assess ColPS	Actions, response times, correctness of item responses	Confirmatory factor analysis	Of	Collaborative problem solving (PISA 2015)	Dependencies among action, time-on task, and performance indicators do not only exist at the construct but also the item (residual) level
Lindner et al.	Time-on task to identify rapid guessing	Correctness of item responses, response times	Latent class analysis	Of	Science achievement	Response times can provide information about rapid-guessing behavior and its relations to cognitive resources and test-taking effort
Naumann	Time-on task data of reading	Correctness of item responses, response times	Linear mixed modeling	Of	Reading literacy (PISA 2009)	Response times can help identify relations between item difficulties, strategic knowledge, skills, and motivation to ultimately craft a validity argument
Simmering et al.	Assessment of non-cognitive skills	Continuous process data (e.g., behavioral, physiological)	–	–	Non-cognitive skills	Challenges and limitations in using technology-enhanced assessments require consideration
von Davier et al.	Data paradigms for educational learning and assessment systems	Response behavior, test content, instructional content	e.g., machine learning	Of/For	Divers	The concept of the “data cube” can be used to label, collect and store data
**Focus on innovative item designs**						
Arieli-Attali et al.	Learning design	Learners' responses and use of learning support	e.g., hidden Markov modeling	For	Divers	The traditional evidence centered design models can be expanded to assess learning
Cipresso et al.	Assessment of unilateral spatial neglect	Correctness of item responses	–	–	Unilateral spatial neglect	Complex 3D environments on mobile devices are promising for the ecological assessment of unilateral spatial neglect
de-Juan-Ripoll et al.	Assessment of risk taking	Behavioral and physiological responses	–	–	Risk taking	Virtual realities (VR) can be employed to simulate hazardous situations realistically
den Ouden et al.	Computerized dynamic assessment of text comprehension skills	Correctness of item responses	Linear modeling and MTMM	For	Text comprehension	Computer-based dynamic assessments bear the potential to support students in acquiring reading skills
Horbach and Zesch	Automated content scoring	Written text	Machine learning	Of	Diverse	Automated content scoring approaches can take into account the variance in learner answers
Kroeze et al.	Automated feedback generation	Written text, actions, correctness of item responses	Descriptive approach, linear model	Of/For	Scientific inquiry in economics and physics	Automated feedback on scientific hypotheses can agree with human ratings to a great extent, and students who receive it are likely to develop better hypotheses than those who don't
**Focus on innovative test designs**						
Born et al.	Computerized adaptive testing and test equating	Correctness of item responses	Item response theory	Of	–	Equating designs and CAT can be combined through a continuous calibration strategy
Csapó and Molnár	Assessment for teaching and learning	Correctness of item responses	Item response theory	For	Mathematics, science, and reading	Teaching and learning can be supported on a large scale by online assessment solutions (authoring, assembly, scoring, delivery, feedback)
Molnár and Csapó	Computerized assessment of cognitive development	Correctness of item responses	Confirmatory factor analysis and structural equation models	Of/For	Mathematics, science, and reading competence	Computerized assessments can capture differences in the academic performance on tests in mathematics, science, and reading across grade levels and make visible the psychological dimension of learning
Rausch et al.	Embedded experience sampling for assessing non-cognitive skills	Survey responses, correctness of item responses	MTMM, item response theory	Of	Non-cognitive facets of problem solving	Embedded experience sampling provides an approach to assess non-cognitive facets of competences through multiple self-reports
Zhang et al.	Computerized adaptive testing of Internet addiction	Survey responses	Item response theory	Of	Internet addiction	A computerized adaptive test of Internet addiction assessed the construct accurately and efficiently, and provided evidence for both the reliability and validity of the resultant test scores
**Focus on statistical approaches**						
Han et al.	Data mining using random forests to predict item performance	Actions, response times, correctness of item responses	Tree-based model	Of	Problem solving (PISA 2012)	A random forest algorithm can generate and select features from the process data that predict students' item responses
He et al.	Text mining and item response data to identify PTSD	Written text, survey responses	Item response theory and text classification	–	Post-traumatic stress disorder	Combining text classification and item response theory models provides an efficient approach to estimating the latent trait
Shin et al.	Topic modeling for item distractor generation	Written text	Machine learning	Of	Knowledge and skills in biology	Latent topic modeling supports the identification of students' misconceptions in biology and aids the development of distractors

Regarding the future of technology-based assessment, we assume that inferences about the individual's or learner's knowledge, skills, or other attributes will increasingly be based on empirical (multimodal) data from less- or non-standardized testing situations. Typical examples are stealth assessments in digital games (Shute and Ventura, 2013; Shute, 2015), digital learning environments (Nguyen et al., 2018), or online activities (Kosinski et al., 2013). Such new kinds of unobtrusive, continuous assessments will further extend the traditional assessment paradigm and enhance our understanding of what an item, a test, and the empirical evidence for inferring attributes can be (Mislevy, 2019). Major challenges lie in the identification and synthesis of evidence from the situations the individual encounters in these non-standardized settings, as well as in validating the interpretation of derived measures. This Research Topic provides much input for these questions. We hope that you will enjoy reading the contributions as much as we did.

## Author Contributions

All authors listed have made a substantial, direct and intellectual contribution to the work, and approved it for publication.

### Conflict of Interest

The authors declare that the research was conducted in the absence of any commercial or financial relationships that could be construed as a potential conflict of interest.
